# Quantification of microfibre release from textiles during domestic laundering

**DOI:** 10.1007/s11356-023-25246-8

**Published:** 2023-01-21

**Authors:** Alice Hazlehurst, Lucy Tiffin, Mark Sumner, Mark Taylor

**Affiliations:** University of Leeds, Leeds, UK

**Keywords:** Microfibres, Textiles, Laundry, Water pollution, Microplastics

## Abstract

Domestic laundering of textiles is being increasingly recognised as a significant source of microfibre pollution. Reliable quantification of microfibre release is necessary to understanding the scale of this issue and to evaluate the efficacy of potential solutions. This study explores three major factors that influence the quantification of microfibres released from the domestic laundering of textiles: test methodologies, laundering variables, and fabric variables.

A review of different test methods is presented, highlighting the variation in quantification created by using different methodologies. A reliable and reproducible method for quantifying microfibre release from domestic laundering is used to explore the impact of laundering and fabric variables experimentally. The reproducibility and reliability of the method used was validated through inter-laboratory trials and has informed the development of European and international testing standards. Our results show that increasing the wash liquor ratio and wash agitation results in a greater mass of microfibres released, but we found that fabric variables can have a greater influence on microfibre release than the laundering variables tested in this study. However, no single fabric variable appeared to have a dominant influence.

Using the data obtained and assumptions for washing load size and frequency, results were scaled to reflect possible annual microfibre release from untreated domestic laundering in the UK. Depending on different laundering and fabric variables, these values range from 6490 tonnes to 87,165 tonnes of microfibre discharged in the UK each year.

## Introduction

The domestic laundering of textile fabrics is considered to be a significant source of microfibre marine pollution (Carney Almroth et al. 2018). Due to their small size and ubiquity, microfibres have increased potential for ingestion by marine organisms (Galloway et al. 2017; Murano et al. 2022; Song et al. 2019; Walkinshaw et al. 2022; Watts et al. 2015; Woods et al. 2018), and their large surface area: volume ratio is thought to increase adverse environmental impacts of microfibres compared to other forms of micro-scale pollutants (Gaylarde et al. 2021; Hurley et al. 2017; Kutralam-Muniasamy et al. 2020; Liu et al. 2021; Rebelein et al. 2021). Microfibres are also expected to easily migrate from the soil due to their low density (Brahney et al. 2020), and be readily transported by ground water and by air, making it unsurprising that their presence has been detected even in very remote areas (Napper et al. 2020). Additionally, even when domestic effluent undergoes waste water treatment, filtration may not be effective at retaining all small fibres and particles, dependent on the type and complexity of the treatment plant (Palacios-Mateo et al. 2021; Ziajahromi et al. 2017). All of which makes it particularly important to quantify the scale of microfibre pollution from domestic laundry to the environment. Furthermore, quantification of microfibre release from laundry is important in the creation and assessment of effective mitigation strategies to reduce this form of pollution.

A large number of studies have attempted to quantify microfibre pollution from domestic laundry via results from laboratory-based experiments. However, many of these rely on a wide range of assumptions or very different methodological approaches, including differences in the laundering devices and approach to the filtration of test liquor and analysis of the microfibres obtained. As the reliability and reproducibility of many of these methods is not well established, generating an acceptable estimate of microfibre release is difficult, particularly as comparisons between many of the studies are not possible because of discrepancies in their approaches to quantification.

In addition to the lack of method consistency, previous studies have used a wide range of laundering variables when assessing microfibre release. For example, washing temperatures tested ranged from 15 (Kelly et al. 2019; Lant et al. 2020) to 80 °C (Hernandez et al. 2017) and wash durations also varied from under 20 min in some studies (Belzagui et al. 2019; Frost et al. 2020; Kelly et al. 2019; Pirc et al. 2016; Vassilenko et al. 2021; Yang et al. 2019; Zambrano et al. 2021,2019), to 90 min and over in others (Dalla Fontana et al. 2021,2020; De Falco et al. 2020,2019,2018; Hernandez et al. 2017; Periyasamy 2021). Variables such as temperature, duration, and agitation during laundering, all influence the quantity of microfibre release, and as there is no agreed standard for these variables, estimates of microfibre release from different wash conditions vary hugely.

A wide variety of different fabrics have also been used between studies, often with little detail about the fabric characteristics being provided, making it difficult to infer the influence of these factors on the quantity of microfibres released during laundering. Finally, many studies have focussed on measuring only synthetic microfibre release, particularly polyester (Browne et al. 2011; Cai et al. 2020; Choi et al. 2021; Dalla Fontana et al. 2020, 2021; Dubaish and Liebezeit 2013; Jönsson et al. 2018; Kelly et al. 2019; Özkan and Gündoğdu 2021; Pirc et al. 2016; Raja Balasaraswathi and Rathinamoorthy 2021; Rathinamoorthy and Raja Balasaraswathi 2021), and understanding of microfibre release for other fibre types, including natural fibres, has not been well explored.

This paper represents a review of current estimates of microfibre release by considering the different methods used to quantify their release. The paper also explores the impact of laundering variables and fabric characteristics on the release of microfibres from domestic laundering. Our testing was performed according to the method described by Tiffin et al. (2021). The reliability of this method was established through extensive inter-laboratory trials, and validation testing confirmed that over 99% of microfibre released in laundering was effectively captured (Tiffin et al. 2021). Using the results of this research, a series of indicative estimates for microfibre release from UK domestic laundering are presented for a number of different scenarios.

## Literature review

Searching SCOPUS for published literature relating to textile microfibres released during domestic laundering returned close to 100 results (search originally conducted in January 2022). From this, papers which focused specifically on the quantification of microfibre release (including the development of methods for quantification) from domestic laundering were selected. A total of 36 papers were included in the review.

### Influence of testing methodologies

Several studies have attempted to quantify microfibre release from domestic laundering; a summary of methodologies used, important testing variables, and estimated microfibre release quantities given by the studies reviewed, is provided in Table 1. Direct comparison of findings between studies is challenging due to the differences in methodologies and sampling (Acharya et al. 2021; Cai et al. 2020; Galvão et al. 2020; Gaylarde et al. 2021; Vassilenko et al. 2021), and as a result, estimated microfibre release ranges from a few thousands (Browne et al. 2011) to several millions of fibres per wash (De Falco et al. 2019,2018; Kärkkäinen and Sillanpää 2020; Kelly et al. 2019; Periyasamy 2021; Vassilenko et al. 2021) depending on the method used.

**Table 1:** Measurement of fibre loss from domestic laundering: literature comparison (NS = not stated, N/A = not applicable)

Author	Test Method	Temp. (°C)	Time (mins)	Agitation (no. ball bearings)	Liquor ratio	Reported microfibre release
(Browne et al. 2011)	Washing machines	40	NS	N/A	NS	> 1900 particles/wash
(Dubaish and Liebezeit 2013)	NS	NS	NS	NS	NS	220–260 mg/garment/wash
(Karlsson 2015)	Washing machines	40	NS	N/A	NS	209,000 fibres/litre
(Napper and Thompson 2016)	Washing machines	30, 40	75	N/A	NS	Poly-cotton: 137,951 fibres/6 kg wash Polyester: 496,030 fibres/6 kg wash Acrylic: 728,789 fibres/6 kg wash
(Pirc et al. 2016)	Washing machines	30	15	N/A	NS	11,300 particles/500 g of fabrics or 6 mg/500g fabric
(Hartline et al. 2016)	Washing machines	30, 40	30, 48	N/A	NS	Average 1174 mg/wash
(Hernandez et al. 2017)	Laboratory method	25, 40, 60, 80	14, 60, 120, 240, 480	10	7:1 l/m^2^	Average approx. 0.025 mg/g
(Sillanpää and Sainio 2017)	Washing machines	40	75	N/A	NS	Polyester: 223,000 particles/wash or 340 mg/wash Cotton: 973,000/wash or 809 mg/wash
(Carney Almroth et al. 2018)	Laboratory method	60	30	25	13:1 l/m^2^	Fleece: 110,000 fibres/garment/wash Knit: 900 fibres/garment/wash
(De Falco et al. 2018)	Laboratory method	40, 60	45, 75, 90	0, 10, 20	NS	Polyester: > 6,000,000 fibres/5 kg wash
(Jönsson et al. 2018)	Laboratory method	40	60	25	5:1 l/m^2^	N/A Focus on method development and validation
(Belzagui et al. 2019)	Washing machines	Ambient	15	N/A	NS	Polyester-elastane: 175 fibres/g/wash or 30,000 fibres/m^2^/wash Acrylic-polyamide: 560 fibres/g/wash or 465,000 fibres/g/wash
(De Falco et al. 2019)	Washing machines	40	107	N/A	NS	Polyester: 640,000–1,100,000 fibres/garment/wash Poly-cotton-modal: 1,500,000 fibres/garment/wash
(Haap et al. 2019)	Laboratory method	40	30	50	6:1 l/m^2^	N/A Focus on method development and validation
(Kelly et al. 2019)	Washing machines and laboratory method	30, 15	60, 15	NS	120:1 l/m^2^ and 240:1 l/m^2^	Standard wash (with detergent): 663,523 fibres/kg/wash Delicate wash (with detergent): 1,474,793 fibres/kg/wash
(Yang et al. 2019)	Washing machines	30,40, 60	15	N/A	17:1 l/m^2^	Acetate: up to 74,816 fibres/m^2^/wash Polyester: up to 72,130 fibres/m^2^/wash
(Zambrano et al. 2019)	Laboratory method	25, 44	16	25	15:1 l/m^2^	Cellulose-based fabrics: 0.2–4 mg/g Polyester: 0.1–1 mg/g
(Cai et al. 2020)	Laboratory method	40	45	0, 10, 20	37.5:1 l/m^2^ and 60:1 l/m^2^	210–72,000 fibres/g/wash
(Cesa et al. 2020)	Washing machines	24	20	N/A	NS	49.8–307.8 mg/wash 18,400–69,600 fibres/wash
(Cotton et al. 2020)	Washing machines	25, 40	30, 85	N/A	NS	Increased microfibre release for 40 °C, 85 min wash compared to 25 °C, 30 min wash
(Dalla Fontana et al. 2020)	Washing machines	40, 30	90, 43	N/A	NS	Delicate/silk cycle: 33.86 mg/kg Cotton cycle: 40.19 mg/kg
(De Falco et al. 2020)	Washing machines	40	107	None	NS	Poly-cotton: 3898 fibres/g/wash Polyester: 709–1747 fibres/g/wash
(Frost et al. 2020)	Laboratory method	20	16	25	NS	Reduced shedding for 70% recycled polyester compared to 40% polyester (not significantly different from virgin polyester)
(Galvão et al. 2020)	Washing machines	20, 30, 40, 60	NS (automatic)	N/A	NS	Average total MF per wash: 297,400 MF/l (83% from cotton fibres) Estimate for 6 kg wash: 18,000,000 MF/6 kg synthetic fibres 1842–6259 mf/g/wash
(Kärkkäinen and Sillanpää 2020)	Washing machines	40	75	N/A	NS	First wash: 100,000–6,300,000 fibres/kg/wash Fifth wash: 19,000–190,000 fibres/kg/wash
(Lant et al. 2020)	Washing machines	40, 15	85, 30	N/A	NS	Approx. 357 mg/3.13 kg wash 96% released fibres = natural (cotton, wool, viscose), 4% = synthetic (acrylic, nylon, polyester)
(Praveena et al. 2020)	Washing machines (household study)	Room temperature	41–60	N/A	NS	0.0069–0.183 g/m^3^ Average: 0.068 g/m^3^
(Celi̇k 2021)	Washing machine	40	53	N/A	NS	3.28–3.91 mg/l/wash 13.1–15.66 mg/kg/wash
(Choi et al. 2021)	Washing machine	40, 20	80	N/A	NS	Hard twist filament: 51.6 ppm Non-twist filament: 88.7 ppm Spun yarn: 107.7 ppm
(Dalla Fontana et al. 2021)	Washing machine	40	90	N/A	NS	38.6–67.9 mg/kg
(Özkan and Gündoğdu 2021)	Laboratory method	40	45	10	37.5:1 l/m^2^	Recycled polyester: 368,094 fibres/kg/wash Virgin polyester: 167,436 fibres/kg/wash
(Periyasamy 2021)	Washing machine	30, 45, 60	60, 75, 90	N/A	NS	2,305,395–4,874,323 fibres/kg/wash
(Raja Balasaraswathi and Rathinamoorthy 2021)	Laboratory method	30	45	10	6.67:1 l/m^2^	Open edge: 72.37 fibres/cm^2^/wash or 0.0239 mg/cm^2^/wash Finished edge: 18.07 fibres/cm^2^/wash or 0.0034 mg/cm^2^/wash
(Rathinamoorthy and Raja Balasaraswathi 2021)	Laboratory method	30	45	5, 10, 15	2.22:1 l/m^2^, 4.44:1 l/m^2^, 6.67:1 l/m^2^, 8.89:1 l/m^2^	Machine wash: 18.06 fibres/cm^2^ Gentle hand wash: 17.33 fibres/cm^2^ Intense hand wash: 23.7 fibres/cm^2^
(Tiffin et al. 2021)	Laboratory method	40	45	50	15:1 l/m^2^	N/A Focus on method development and validation
(Vassilenko et al. 2021)	Washing machine	41	18	NA	136.4:1 l/kg	8,809–> 6,877,000 fibres/wash 9.6–1240 mg/kg/wash
(Zambrano et al. 2021)	Laboratory method	25	16	25	NS	9000-14,000 particles/g/wash Softener/durable press-treated cotton: 1.30–1.63 mg/g/wash Untreated cotton: 0.73 mg/g

Several important methodological factors can influence the quantity of microfibre release from a sample in these laboratory tests, including but not limited to: the type of laboratory method, for example, testing in a domestic washing machine or using a simulated laundering device (Gyrowash or similar); the means of filtering the test liquor, including the filtration method and the type and retention efficiency of the filters themselves; and the metric for quantification, by fibre count or by fibre mass (Tiffin et al. 2021).

To explore the influence of methodological approaches from different studies on the quantification of microfibre release, the results provided in Table 1 were standardised to either fibres/kg or mg/kg of release to allow comparison. Only results which were expressed as per unit mass could be included in this comparison.

An estimation for the annual microfibre release for the UK was calculated for each method using the following assumptions: the average UK wash load is approximately 5 kg, and the average UK household completes 260 wash loads each year (Webber et al. 2016). With 27.8 million households in the UK, according to the Office for National Statistics (ONS) (Sanders 2019), this equates to 7.228 billion wash loads annually. An additional assumption has been made that the results from each study are indicative of a single wash load. However, it should be acknowledged that several other factors, such as the size and fibre mix of the wash load, the age of the items being washed, and the level of soiling will also influence results in real washing conditions. The resultant estimates are provided as a means for comparison, to indicate the scale of variation that may occur as a result of different variables used in the assessment of microfibre release.

**Fig. 1:**
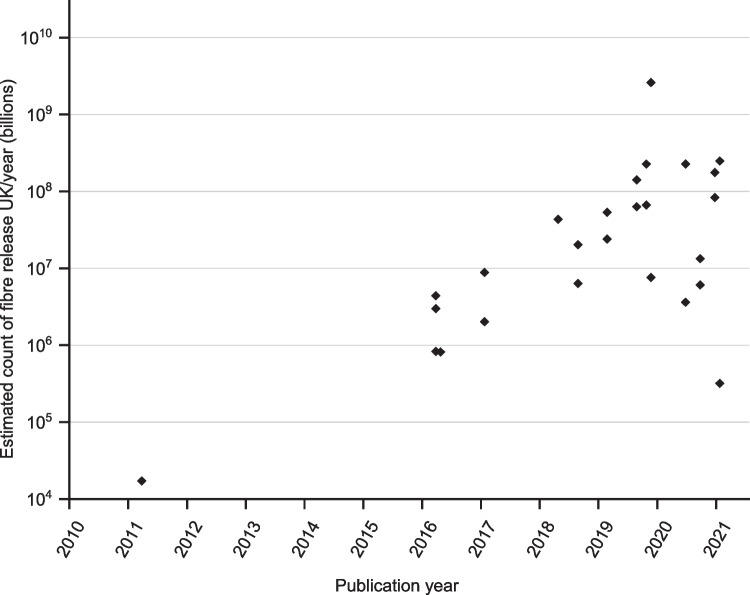
Comparison of the estimated microfibre release count UK/year (billions) based on published microfibre release counts tested by different methodologies (logarithmic scale)

The estimated microfibre release counts are shown in Fig. 1. Multiple data points are provided where authors offered multiple microfibre release results for different conditions as detailed in Table 1. Based on our calculations, estimates range from 17,167 billion microfibres (originally reported as > 1900 particles released from a 4 kg wash load (Browne et al. 2011)) to 2,602,080 trillion fibres (originally reported as 72,000 fibres/g (Cai et al. 2020)) discharged annually for the UK scenario described. The mean of these estimates of annual UK release is 155,097,349 billion microfibres and the range is 2,602,063 trillion fibres. Interestingly, there is an upward trend of the microfibre count reported with more recent studies.

**Fig. 2:**
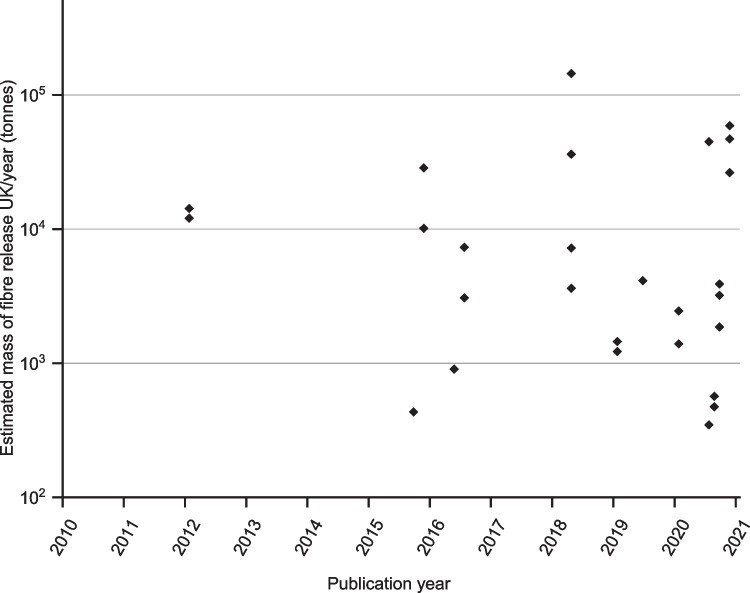
Comparison of the estimated mass of microfibre release UK/year (tonnes) based on the published mass of microfibre release tested by different methodologies (logarithmic scale)

The estimates for the annual mass of microfibre released for the UK scenario are shown in Fig. 2, again with multiple data points provided where authors offered multiple microfibre release results in their original studies. From our calculations, the lowest estimate is 347 tonnes (originally reported as 9.6 mg/kg (Vassilenko et al. 2021)), while the largest mass estimate is 144,560 tonnes (originally reported as 4 mg/g (Zambrano et al. 2019)). The mean estimated mass of annual microfibre release for the UK scenario is 17,234 tonnes and the range is 144,213 tonnes. For comparison, the UK disposes of approximately 350,000 tonnes of clothing of all fibre types to landfill each year (Welden 2019).

There is a huge variation in the estimates for the number and the mass of microfibres being reported. It is clear from this analysis that the discrepancies between methods do not provide confidence in the current estimates for microfibre release from laundering, and there is a clear need for a standardised, reliable method for the useful comparison of results between studies. This is particularly important for assessing the impact of mitigation strategies to reduce microfibre pollution from domestic laundry.

### Influence of laundering variables

There are also a number of important laundering variables to consider which can influence microfibre release. These include wash temperature, wash duration, liquor ratio, washing agitation, and the presence of detergent or other washing products.

The impact of temperature on microfibre release has been explored through several studies with mixed results. Some studies found wash temperature had no significant effect on microfibre release (De Falco et al. 2018; Hernandez et al. 2017; Kelly et al. 2019) while others reported greater release for higher wash temperatures (Cotton et al. 2020; Napper and Thompson 2016; Periyasamy 2021; Yang et al. 2019; Zambrano et al. 2019). This divergence in results may be explained by the use of different textile materials, with different thermal properties, for testing.

Most studies found wash duration had little or no significant influence on microfibre release (De Falco et al. 2018; Hernandez et al. 2017; Kelly et al. 2019). Kelly et al. (2019) found that the same quantity of fibres was released during a 15 min wash as for a 60 min wash, which they suggested might indicate that the majority of microfibre release occurs in the first 15 minutes of the wash cycle.

The level of agitation and friction within the washing process is considered to be a major factor affecting the quantity of microfibre released. Agitation is a function of drum size, drum configuration, rotational speeds, and wash liquor volume to load ratio. Kelly et al. investigated the impact of agitation by considering the effect of rotation speed and wash liquor volume on the quantity of microfibres released from a single laundering cycle (Kelly et al. 2019). They showed increasing rotational speed resulted in a greater release of microfibres, as did increasing the ratio of wash liquor to load. In a separate study, Lant et al. found microfibre release increased when washing smaller loads, theorising that smaller wash loads, with higher wash liquor to load ratios, caused increased flow of the wash liquor through the fabrics which promoted higher microfibre release (Lant et al. 2020). Hartline et al. also noted the influence of agitation; they found greater release of microfibres from a top-loading washing machine compared to front-loading, which they suggested was due to the central agitator of a top-loading machine having a more abrasive effect compared to the rotating drum of a front-loading machine (Hartline et al. 2016). Rathinamoorthy and Raja Balasaraswathi also found a positive correlation between increasing agitation and microfibre release with increasing number of ball bearings (5, 10, 15) in their laboratory device method (Rathinamoorthy and Raja Balasaraswathi 2021). On the other hand, Cai et al. found no significant difference from increasing the number of ball bearings from 0 to 20 (Cai et al. 2020). The conflicting results could be explained by differences between the two testing methods and different fabrics being tested.

In full-scale domestic washing machines when washing loads are small relative to the drum capacity, there tends to be more movement between garments and the drum, and between individual garments. Where the load is large relative to the drum capacity, there is less movement and therefore, less friction (Mac Namara et al. 2012; Yun and Park 2015). This suggests that microfibre release could well have been overestimated in studies where only a single garment or fabric specimen was laundered individually in a full-scale domestic washing machine due to increased agitation compared to typical washing loads.

Where studies included detergent in their laundry testing, several groups noted the detergent was a significant contaminant when analysing microfibre release. Clogging or “caking” of detergent on the filters, particularly when powder detergents were used, interfered with the analysis making it difficult to differentiate between microfibres and residual detergent, and therefore, difficult to quantify microfibre release from these studies (De Falco et al. 2018; Hernandez et al. 2017; Jönsson et al. 2018; Yang et al. 2019; Zambrano et al. 2019).

For those studies that could exclude significant errors due to detergent contamination, the impact of detergents on microfibre release remains unclear. Some studies reported no significant impact of detergent (Kelly et al. 2019; Lant et al. 2020; Napper and Thompson 2016; Pirc et al. 2016), while others reported increased microfibre release when detergent was present (Carney Almroth et al. 2018; De Falco et al. 2018; Hernandez et al. 2017; Periyasamy 2021; Yang et al. 2019; Zambrano et al. 2019). It appears from such studies that powder detergent had a greater impact on microfibre release than liquid detergent (De Falco et al. 2018; Hernandez et al. 2017; Periyasamy 2021). It has been suggested that the higher microfibre release due to powder detergents might be explained by increased friction between small insoluble particles within the powder formulation that penetrate into the fabric, leading to fibre damage and thus microfibre fragmentation (De Falco et al. 2018). Of the studies reviewed, only Cesa et al. found that the use of detergent reduced microfibre release (Cesa et al. 2020). However, this negative correlation was only observed for synthetic garments, with cotton test samples demonstrating higher microfibre release overall, possibly suggesting that the influence of fabric composition was more dominant than the detergent used in this case.

Several researchers have reported a reduction in microfibre release with increasing number of repeat wash cycles (Belzagui et al. 2019; Cai et al. 2020; Carney Almroth et al. 2018; Cesa et al. 2020; Kelly et al. 2019; Lant et al. 2020; Özkan and Gündoğdu 2021; Pirc et al. 2016; Rathinamoorthy and Raja Balasaraswathi 2021; Sillanpää and Sainio 2017; Vassilenko et al. 2021; Zambrano et al. 2019). This suggests that a majority of the microfibres available for release are lost from the fabric structure in the first few washing cycles. Only Dalla Fontana et al. found no significant reduction in microfibre release across multiple washes (up to 5 cycles), but they noted that this could be due to loose fibres having already become detached during the more intensive pre-test scouring procedure used in their method (Dalla Fontana et al. 2021). It should be noted that in the studies reviewed, fabric specimens were not subjected to any additional physical ageing between each wash. In real-life situations, everyday use and wear of textile items will disturb the fabric surface, potentially mobilising and fragmenting fibres that are held within the fabric structure. With subsequent washing these fragments may be released into the wash liquor, resulting in increased microfibre release over the lifetime of a garment.

### Influence of fabric variables

Different fabrics can be expected to differ in their microfibre release dependent on their characteristics and properties. Very few studies have been dedicated to understanding the influence of fabric characteristics on microfibre release, and there is little consensus in the data currently available.

Raja Balasaraswathi and Rathinamoorthy provide the most comprehensive investigation of the influence of fabric characteristics and properties to date; they found that fabric parameters such as stitch density, tightness, thickness, and filament denier were of greater influence, and provided a better indication of microfibre release than physical fabric properties such as bursting strength and pilling resistance (Raja Balasaraswathi and Rathinamoorthy 2021). They also noted increased microfibre release with increased mass per unit area and fabric thickness, owing to an increase in fibres per unit area available for detachment and release (Raja Balasaraswathi and Rathinamoorthy 2021). However, De Falco et al. found mass per unit area to have little influence (De Falco et al. 2018). Some researchers have noted the influence of the tightness of the fabric structure, with tighter, more compact structures deemed favourable to reduce mobility and release of fibres (De Falco et al. 2019; Raja Balasaraswathi and Rathinamoorthy 2021; Yang et al. 2019).

The influence of some yarn types on microfibre release was explored by Choi et al. who found that, for the same woven construction, fabrics constructed from staple spun yarns released more fibre than filament yarns. Fibres in a staple spun yarn are shorter and have greater mobility than filament yarns, they are more vulnerable to release when the fabric is agitated so they are expected to release more microfibres. Filament yarns with no twist shed more than highly twisted yarns (Choi et al. 2021). The higher release from non-twist filaments is theorised to be due to reduced inter-fibre friction leading to greater fibre freedom and therefore greater potential for fibre damage (Choi et al. 2021). Zambrano et al. theorised that the generation of microfibres is largely a function of pill formation and that the formation of fuzz would be highly influential to microfibre release (Zambrano et al. 2019). However, Dalla Fontana et al. found they could not correlate pilling performance with microfibre release, as fabrics which performed poorly in pilling testing had more pills held at the fabric surface which were not released during laundering (Dalla Fontana et al. 2021). There is a clear need for greater understanding of the influence of different fabric characteristics on microfibre release as current data is limited and findings are often contradictory.

This research explores the influence of laundering variables on microfibre release by systematically comparing different in-wash conditions. The influence of fabric characteristics is also explored by testing a range of commercially available fabric types. A reliable and reproducible test method is employed throughout to eliminate the influence of different testing methodologies. The results of this research have been scaled to provide an indicative UK annual microfibre release for the purposes of comparison with results from the existing literature.

## Methods and materials

A range of experiments was carried out to explore the influence of laundering and fabric variables on estimates for microfibre release. The experimental component of the research used the laundering test method detailed by Tiffin et al. (2021), which is briefly described below. The method utilises a Gyrowash simulated laundering device which is used extensively within the textile industry to approximate the abrasive action of domestic laundering (American Association of Textile Chemists and Colorists 2013; British Standards Institution 2010). The reproducibility of the method has been validated through inter-laboratory trials, and it forms the basis for the AATCC (American Association of Textile Chemists and Colorists) testing standard, *Test Method for Fiber Fragment Release During Home Laundering* (American Association of Textile Chemists and Colorists 2021), as well as European and international testing standards currently under development (International Organization for Standardization 2022).

### Laundering tests

All laundering tests were conducted using a simulated laundering method. This approach was selected as a simulated laundering device offers better control over in-wash factors such as duration, temperature, liquor ratio and agitation, allowing for much more reliable comparison between test conditions. The consistency and reproducibility of in-wash factors in domestic washing machines is inadequate for such comparisons, especially where “fuzzy machine logic” is employed as these operations will be continually adjusted throughout the washing cycle, meaning that no two cycles will be the same. Using a Gyrowash device also ensures the collection of all microfibres released as simulated laundering occurs in a closed canister. The efficacy of microfibre collection by this method was validated as over 99% by Tiffin et al. (2021).

For each test, a minimum of 8 replicate specimens were prepared with hemmed edges to prevent any fibre release from the cut edge. Each specimen was oven dried at 50 °C for a minimum of 4 h and their masses were recorded to an accuracy of 0.0001 g prior to testing (Adam Equipment Co. Ltd, ADA 210 Balance, Capacity: 210 g, Readability: 0.0001g). Test specimens were laundered in a Gyrowash simulated laundering device (James Heal Gyrowash 1615/8, James Heal, UK) with the rotational speed of 40 rpm, using 1200 ml capacity stainless steel canisters. Specimens were laundered in distilled water only, without the addition of detergent or any other laundering auxiliaries. The testing parameters—temperature, duration, liquor ratio, and mechanical agitation (provided by stainless steel ball bearings)—were altered dependent on the test (see “Laundering variables” and “Fabric variables” Sections).

Resultant test liquors from each canister were subjected to a vacuum-assisted single-stage filtration process (16309 All-glass vacuum filter holder, Sartorius Stedim, Germany) using 1.6 μm glass microfibre filters (Whatman Grade GF/A Glass Microfiber Filters) to capture released microfibres. The oven-dry mass pre- and post-filtering (oven dried at 50 °C for a minimum of 4 h) were used to determine the mass of microfibre released. This was normalised to the mass of the original fabric specimen and expressed as mg/kg. The results provided represent the mean microfibre release in mg/kg and the error reported is the 95% confidence interval.

**Fig. 3:**
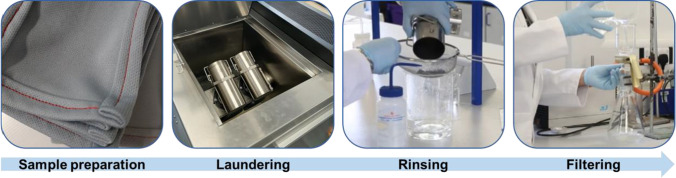
Laundering test method procedure

A visual summary of the procedure is provided in Fig. 3.

### Laundering variables

To assess the influence of washing variables on the mass of microfibre released, a number of important variables were selected for investigation. These included wash temperature (40 °C and 90 °C), wash duration (30 min and 60 min), liquor ratio (7.5:1 l/m^2^ and 15:1 l/m^2^), and mechanical agitation (10, 20, 40, and 50 ball bearings). Each of these variables was altered in turn to assess their influence independently of the other variables. The conditions used in each test are provided in Table 2. For each condition, 8 replicate specimens were tested. A single fabric quality (100% polyester, knitted fleece, 225 g/m^2^) was used for each washing variable test to ensure fabric variations did not influence results for this part of the testing. Fabric specimens were cut to 240 mm × 100 m and the edges were overlocked and lock-stitched to prevent any fibre release from the cut edge. Laundering testing was completed according to the method described above. Results for temperature, wash duration, and liquor ratio were compared using a two-tailed *t*-test, and results for agitation were compared using a one-way ANOVA with post-hoc Tukey test to assess the significance of any differences in microfibre released.

**Table 2:** Laundering variables testing - conditions of test (8 replicates in each condition)

	Temperature (°C)	Duration (mins)	Liquor ratio (l/m^2^)	No. ball bearings
Temperature trial	40, (90)	60	15:1	50
Duration trial	40	60, (30)	15:1	50
Liquor ratio trial	40	60	15:1, (7.5:1)	50
Agitation trial	40	60	15:1	50 (10, 20, 40)

The impact of repeat washings on the mass of microfibre released was also assessed using the baseline wash variables described by Tiffin et al. (2021): temperature, 40 °C; duration, 60 min; liquor ratio, 15:1 l/m^2^ (360 ml per specimen); mechanical agitation, 50 stainless steel ball bearings. 8 replicate fabric specimens were prepared for this test and were oven dried at 50 °C for a minimum of 4 h before the first wash (as per the standard method described above), and again between each of the repeated, identical 5 washes. The results for repeat washings were compared using a single-factor ANOVA with post-hoc Tukey test to assess the significance of any differences in microfibre released.

### Fabric variables

To assess the influence of fabric variables on the mass of microfibre released, a selection of 16 commercially available fabrics with varied specifications were sourced for testing as detailed in Table 3. The fabrics represent a range of fabrics commonly used in clothing for the UK market. A broad range of fabric characteristics has been included for consideration, including fibre compositions (polyester, cotton, viscose, and blends), yarn types (staple and filament), and fabric constructions (knitted and woven). Fabric specimens were cut to 290 × 150 mm and the edges were double-hemmed and lock-stitched to prevent any fibre release from the cut edge (resultant specimen size 240 × 100 mm). For each test, 24 replicate specimens were prepared (with the exception of PET_1 which had 8 replicates due to experimental complications). Laundering testing was completed according to the method described above, and for this part of testing, the laundering variables were kept constant—temperature 40 °C; duration, 45 min; liquor ratio, 15:1 l/m^2^ (360 ml per specimen); mechanical agitation, 50 stainless steel ball bearings—to ensure that laundering variables did not influence results.

**Table 3:** Fabric characteristics testing - specification overview (24 replicates in each condition (excepting PET_1 with 8 replicates))

Fabric reference	Fibre composition	Fibre/yarn characteristics	Fabric structure	Fabric weight (gm^2^)	Mechanical finishing
PET_1	100% Polyester	Staple, chenille, 150 D	Knit, double jersey jacquard, 3 gg	250	-
PET_2	100% Polyester	Filament, 100 D	Knit, jersey grid fleece, 24 gg	175	-
PET_3	100% Polyester	Filament, 75 D	Knit, pique interlock, 28 gg	125	-
PET_4	100% Polyester	Filament, 70 D/50 D	Knit, circular, 36 gg	85	-
PET_5	100% Polyester	Filament, 30 D	Weave, ripstop	56	-
PET-ELA_1	84% Polyester/16% Elastane	Filament/ Filament, 75D/40 D	Knit, single jersey fleece, 32 gg	185	Brushed back/Peached face
PET-ELA_2	95% Polyester/5% Elastane	Filament, 100 D	Knit, jersey, 28 gg	165	-
PET-ELA_3	92% Polyester/8% Elastane	Filament, 75 D/50 D	Knit, single jersey fleece, 24 gg	154	Brushed
PET-VIS	65% Polyester/35% Viscose	Staple, 2/24 Nm/2/25 Nm	Weave, 2x1 twill	265	-
PET-VIS-ELA	75% Polyester/23% Viscose/2% Elastane	Staple, 25s/20 D/40 D	Weave, twill	240	Relax dry
rPET-TEN-ELA	61% rPET/34% Tencel/5% Elastane	Filament/ Staple 75 D/ 70s	Knit, tech fleece, 28 gg	250	Brushed
MER-PET	47% Merino/53% Polyester	Staple/ Filament, 70 D	Knit, circular, 30 gg	125	-
PET-COT_1	65% Polyester/35% Cotton	Filament	Weave, plain	194	Peached face
PET-COT_2	65% Polyester/35% Cotton	Filament	Weave, plain	184	-
COT	100% Cotton	Staple, 30s	Knit, fleece, 20 gg	360	Brushed back
NYL	100% Nylon	Filament, 20 D/20 D	Weave, taffeta	38	-

## Results and discussion

### Influence of laundering variables

**Fig. 4:**
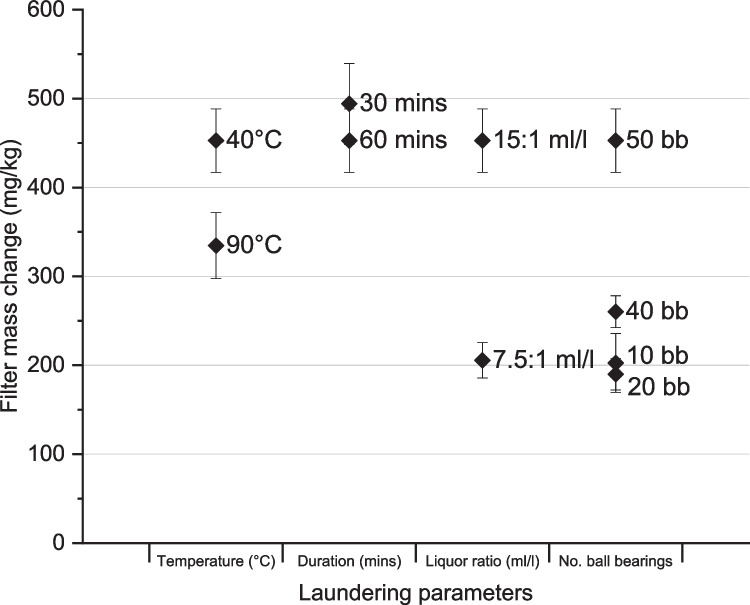
Comparison of the microfibre release under different laundering conditions: temperature, duration, liquor ratio, agitation (error bars represent 95% confidence interval)

Results for the mass of microfibre released during the laundering variables testing are presented in Fig. 4.

Increasing the wash temperature from 40 to 90 °C reduced the microfibre release by 26% (from 453 ± 36 to 335 ± 37 mg/kg, t(14) = 0.00, *p* = 0.05). These results differ from some of the existing literature wherein several studies found that wash temperature had no significant effect on microfibre release (De Falco et al. 2018; Hernandez et al. 2017; Kelly et al. 2019), while others reported higher microfibre release for higher wash temperatures (Cotton et al. 2020; Napper and Thompson 2016; Periyasamy 2021; Yang et al. 2019; Zambrano et al. 2019). However, it should be noted that other studies tended to use wash temperatures of 25 °C, 40 °C, and 60 °C and did not test at temperatures as high as 90 °C. It is possible that the reduction in microfibre release observed at 90 °C could be due to the wash temperature taking the polyester sample through its glass transition temperature (*T*_g_). The glass transition of polyester typically occurs at between 67 and 81 °C depending on the crystallinity of the polymer (Demi̇rel et al. 2011). At this temperature, the polyester transitions to a more malleable, less brittle structure which may in turn lead to a reduction in the fragmentation of fibres caused by the mechanical action of the laundering process. As the glass transition temperature of other textiles fibres varies, the impact of elevated temperatures on microfibre release may well vary significantly for fabrics of other fibre compositions such as polyamide (*T*_g_ 40–55 °C) (Deopura and Padaki 2015) or acrylic (*T*_g_ 80–90 °C) (Richards 2015). For example, Hernandez et al., the only study to approach the glass transition temperature of polyester, did observe a slight decrease in microfibre release at 80 °C but the decrease was not statistically significant (Hernandez et al. 2017). It is suspected that the glass transition temperature of the polyester used in their study may not have been reached or held at the temperature long enough to have an impact, or the test variables that were included in the study masked the impact of this elevated temperature.

**Fig. 5:**
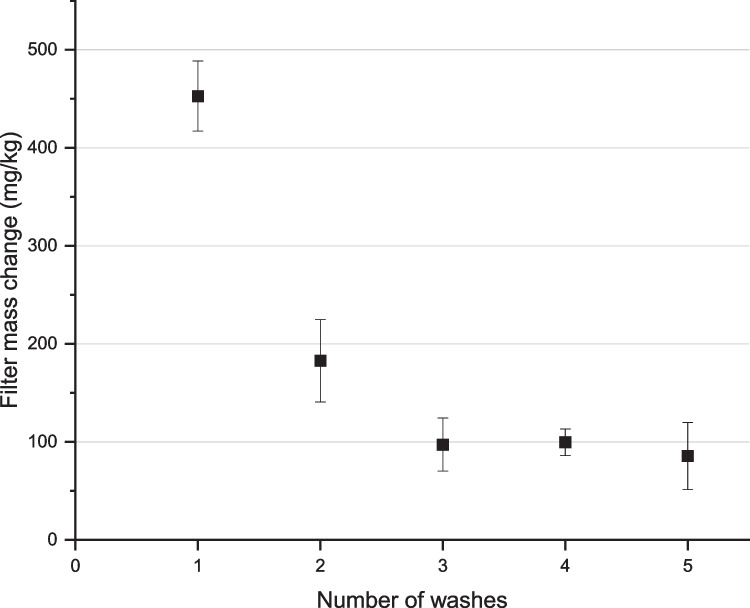
Repeat washings comparison (error bars represent 95% confidence interval)

The impact of wash duration on microfibre release was tested at 30 and 60 min. To test the hypothesis that wash duration affects the amount of microfibre released, a two-tailed *t*-test was performed and revealed that there is no significant difference between the means (494 ± 45 mg/kg at 30 min, and 453 ± 36 mg/kg at 60 min, t(14) = 0.18, *p* = 0.05). This is in line with a majority of other studies which found no significant impact of wash time on microfibre release (De Falco et al. 2018; Hernandez et al. 2017; Kelly et al. 2019). As suggested by Kelly et al. (2019) it is suspected that the majority of microfibres are loosely held in the fabric and yarn structure, and these are released relatively easily and quickly early on in the washing cycle (Kelly et al. 2019). Increasing wash duration, therefore, has little effect on the total release of microfibres. This hypothesis is further supported by the results of the repeat washing cycles shown in Fig. 5.

Unlike wash temperature and wash duration, the liquor ratio was found to have a positive relationship on the mass of microfibre release. More than twice as much material was released from the fabric when the liquor ratio was doubled from 7.5:1 l/m^2^ (206 ± 20 mg/kg) to 15:1 l/m^2^ (453 ± 36 mg/kg) (t(14) = 0.00, *p* = 0.05). This is in line with Kelly et al. (2019) who reported greater microfibre release for larger wash volumes, and Lant et al. (2020) who also found increased mass release when washing smaller loads (wherein the wash liquor to fabric ratio was higher) (Kelly et al. 2019; Lant et al. 2020). It is expected that with higher liquor volumes, there is an increased flow of liquid through the fabric, causing more fibres to be mobilised and/or fragmented from the fabric structure. Based on our results and others’, microfibre release from domestic laundry could be reduced by filling the washing drum, which reduces the wash liquor ratio. However, there may be implications for the safe operation of the washing machine and the washing efficacy for the increased load size.

To mimic the agitation and abrasion experienced by fabrics in domestic washing machines, stainless steel ball bearings are typically added to the Gyrowash canisters. The impact of agitation in domestic laundry on microfibre release can be assessed by altering the number of ball bearings used, with more ball bearings increasing the level of agitation experienced by the fabric sample. Figure 4 shows as agitation increases, with more ball bearings in the canisters, there is an increase in microfibre release (mean releases for 10, 20, 40, 50 ball bearings were 203 ± 33 mg/kg, 190 ± 18 mg/kg, 260 ± 18 mg/kg, and 453 ± 36 mg/kg, respectively). A one-way ANOVA revealed that there was a statistically significant difference in microfibre release between at least two groups (F(3, 28) = [75.51], *p* = 0.00). There was no statistically significant difference in mean microfibre release between 10 and 20 ball bearings (*p* = 0.90) which is in agreement with the findings of Cai et al. (2020). However, Tukey’s test for multiple comparisons found that the mean microfibre release was significantly different between 10 and 40 ball bearings (*p* = 0.03), 10 and 50 ball bearings (*p* = 0.00), 20 and 40 ball bearings (*p* = 0.01), 20 and 50 ball bearings (*p* = 0.00), and 40 and 50 ball bearings (*p* = 0.00). The mass of released material was more than doubled when the number of ball bearings was increased from 10 (203 ± 33 mg/kg) to 50 (453 ± 36 mg/kg). There are two possible mechanisms leading to microfibre release as a result of agitation. Increased agitation causes disruption to the surface of the fabric which mobilises any loose fibres that may be present in the fabric and yarn structures. In addition, the mechanical action created by the agitation increases the likelihood of fibre damage and breakage which could lead to a greater number of fibre fragments being formed and released from the fabric surface.

The results of the repeat washings, where the same samples were washed, oven-dried and re-washed for 5 identical cycles, can be found in Fig. 5. The vast majority of microfibre was released during the first wash (453 ± 36 mg/kg) with a significantly reduced mass of material released from the subsequent washes (183 ± 42 mg/kg, 97 ± 27 mg/kg, 100 ± 14 mg/kg, and 86 ± 34 mg/kg for the second, third, fourth, and fifth wash, respectively). A one-way ANOVA revealed that there was a statistically significant difference in mean microfibre release between at least two groups (F(4, 35) = [90.29], *p* = 0.00). Tukey’s test for multiple comparisons found that the mean microfibre release was significantly different between the first wash and all subsequent washes (*p* = 0.00 in all cases), and also between the second wash and subsequent washes (*p* = 0.001, *p* = 0.001, *p* = 0.00, when comparing wash 2 to wash 3, 4, and 5, respectively). For this fabric, results indicate that the rate of microfibre release plateaued for washes 3–5, and there was no statistically significant difference in mean microfibre release between washes 3, 4, and 5 (*p* = 0.90 in all cases). Other researchers have also reported a decrease in microfibre release after the first wash (Belzagui et al. 2019; Cai et al. 2020; Carney Almroth et al. 2018; Cesa et al. 2020; Kelly et al. 2019; Lant et al. 2020; Özkan and Gündoğdu 2021; Pirc et al. 2016; Rathinamoorthy and Raja Balasaraswathi 2021; Sillanpää and Sainio 2017; Vassilenko et al. 2021; Zambrano et al. 2019) despite differences in methodologies and fabrics tested.

It should be noted that in our work as well as others’, the fabric specimens were not subjected to any additional ageing between each wash. In real-life situations, everyday use and wear of textile items will disturb the fabric surface, loosening and fragmenting fibres held within the fabric structure. With subsequent washing these fragments may be released into the wash liquor, resulting in increased microfibre release over the lifetime of a garment.

### Influence of fabric variables

**Fig. 6:**
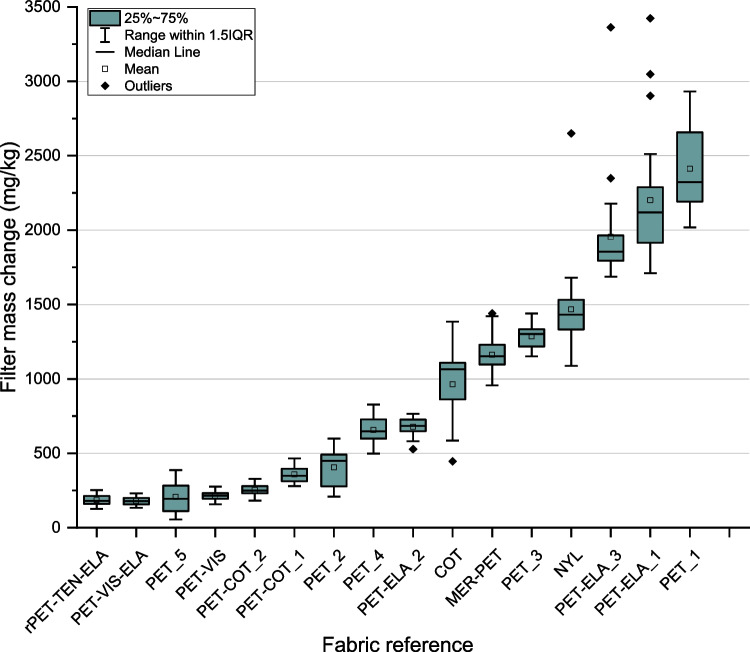
Microfibre release results for fabrics of varied specifications

Results for the mass of microfibre released during the fabric variables testing are presented in Fig. 6. Some level of microfibre release is evident from all fabrics tested and there is a relatively large spread of data, from 180 ± 11 mg/kg for fabric PET-VIS-ELA to 2412 ± 222 mg/kg for fabric PET_1, indicating a wide range of microfibre performance between the different fabrics. As the testing method and laundering variables were kept constant for these tests, the results clearly demonstrate that fabric characteristics are influencing microfibre release. The fabrics selected represent a broad selection of characteristics from fibre to finished fabric level. Textile fabrics typically have a hierarchical structure: fibres are twisted or spun to form yarns which are then knitted or woven into fabrics. Dyeing typically occurs at the yarn or fabric level to imbue colour and chemical and/or mechanical treatments are often applied to modify the surface, wearing properties, or aftercare characteristics of the fabric (Eberle 2008; Sinclair 2014; Taylor 1997).

Despite a clear indication that fabric characteristics play an important role in determining microfibre release, identifying which of the fabric variables have the strongest influence on microfibre release is challenging due to the complexity of textile manufacturing processes.

In terms of fibre type, there is no strong indication of either filament (indefinite, continuous fibre lengths) or staple (short, discrete fibre lengths) yarns having favourable performance for the fabrics tested. It was expected that fabrics constructed of staple yarns would shed more microfibres than those comprised of filament fibres as the short staple fibres could more easily be mobilised from the yarn structure. It is interesting to note that several staple fibre fabrics did not meet this expectation, having very low release rates compared to some filament fibre fabrics. This is likely explained by fabric structure, as a tightly woven construction with smooth and flat surface could help to retain fibres and reduce the likelihood of microfibre release. Conversely, a number of the fabrics tested demonstrated higher release than was expected for filament fabrics; however, this could again be due to differences in structure or finishing technique, especially for brushed fabrics where the filament yarns will have been disrupted or fractured during this processing.

Mechanical finishing does appear to influence microfibre release to some extent although not in all cases. For example, some of the lowest results were from fabrics which had been brushed or peached (typically achieved by disrupting the fabric surface with fine wires to raise the surface and provide a soft handle (Eberle 2008)), which could be expected to have a greater likelihood of broken and damaged fibres at the fabric surface. Fabric PET-COT_1 and PET-COT_2 have the most directly comparable specifications, with the only difference being that PET-COT_1 has been peached on the fabric face, whereas PET-COT_2 has no mechanical finishing. PET-COT_1 exhibited lower microfibre release than PET-COT_2 (252 ± 14 mg/kg and 359 ± 21 mg/kg, respectively), suggesting that mechanical finishing reduced microfibre release in this case.

Overall, these results do demonstrate that there are multiple, related fabric factors which influence microfibre release. It is highly likely that the release of microfibres will not be driven by any one single factor or fabric characteristic, but it will be a combination of characteristics that determine the propensity for microfibre release.

### Estimated UK per annum microfibre release for different laundering conditions and fabric characteristics

Estimates of per annum microfibre release for a UK scenario for the different washing scenarios are shown in Table 4, based on the polyester fleece test fabric. Estimates were calculated using the same assumptions applied to the literature, with the expectation that results obtained from changing conditions using the method described here could be indicative of relative changes in real domestic laundering, These estimations are not intended to present definitive, real-world emission levels, rather, they are intended to allow comparison of results at a relevant scale and provide a more useful indication of the range and scale of change in microfibre release when laundering and fabric variables are altered. At the baseline conditions used in our testing, the estimated annual UK microfibre release from the domestic laundry is 16,384 tonnes. This assumes all the laundry loads contain new, previously unwashed, items. As this figure is based only on the polyester test fabric, it also does not account for microfibre release from other plastic-based fibre types (e.g. nylon, acrylic) or microfibre release from non-plastic fibres such as cotton or wool. Different fibres will have varied propensity for the formation and release of microfibres, therefore estimates of total microfibre release from domestic laundry will be affected by the overall fibre composition of the full domestic laundry load.

**Table 4:** UK per annum microfibre release estimates for different washing scenarios based on the polyester fleece test fabric

Temperature (°C)	Duration (mins)	Liquor ratio (l/m^2^)	No. ball bearings	Mean microfibre release for test specimen (mg/kg)	Estimated microfibre release for 5 kg wash load (g)	Estimated UK annual microfibre release (tonnes)
40	60	15:1	50	453.35	2.27	16,384
90	60	15:1	50	334.03	1.67	12,072
40	30	15:1	50	493.84	2.47	17,847
40	60	7.5:1	50	205.53	1.03	7428
40	60	15:1	10	202.64	1.01	7323
40	60	15:1	20	189.81	0.95	6860
40	60	15:1	40	260.21	1.30	9404

Notably, when reducing the in-wash agitation from the baseline of 50 ball bearings to 10 or 20 ball bearings, our estimate for microfibre release is reduced by more than half, to approximately 7000 tonnes. This suggests that reducing agitation in domestic washing could significantly reduce the UK’s contribution to microfibre pollution. As noted previously, this can be achieved by full washing loads to limit movement and therefore mechanical action during the laundering cycle (Mac Namara et al. 2012; Yun and Park 2015).

The estimates in Table 4 present a worst-case scenario where each wash cycle contains only new, previously unwashed garments. Our repeat washings findings show that microfibre release significantly reduced after the first wash and this has implications for estimates for the total UK microfibre release. If assuming an average domestic wash load for the UK has an equal distribution of items that are new and unwashed, and items that have had one, two, three, four, and five washes, the estimate for annual microfibre release in the UK becomes 6639 tonnes. This estimate is based on repeated washes without additional ageing between each wash cycle which is likely to affect microfibre release in real-life situations.

In addition to wash variables considered in this paper, there are other laundering variables which have not been reported. For example, adding detergent and other washing aids such as softeners might impact microfibre release and add to the complexity for establishing estimations of total release.

If the polyester fleece fabric used as the basis for the results in Table 4 is replaced with other test fabrics, another set of estimates can be calculated. Based on the results for PET_1, an estimate of microfibre release for UK domestic laundering is 87,165 tonnes. The lowest estimate from our fabric testing is 6490 tonnes based on the results for fabric PET-VIS-ELA. As these samples were tested using the baseline wash conditions this compares with 16,384 tonnes for the polyester fleece fabric in Table 4. This demonstrates that the fabric type has a significant influence on estimates for microfibre release, even when laundering conditions are kept constant. As mentioned previously, these estimates represent a worst-case scenario, as they assume a full washing load of previously unwashed items of a single fabric composition and construction. In real domestic washing scenarios, a mix of garments of different fibre types and fabric structures will be being laundered within any given household. As an indication of the possible fibre mix in UK laundering, market research indicates that less than 30% of garments sold in the UK in 2020 were polyester-based, while over 50% were cotton-based (Palmer 2021).

**Fig. 7:**
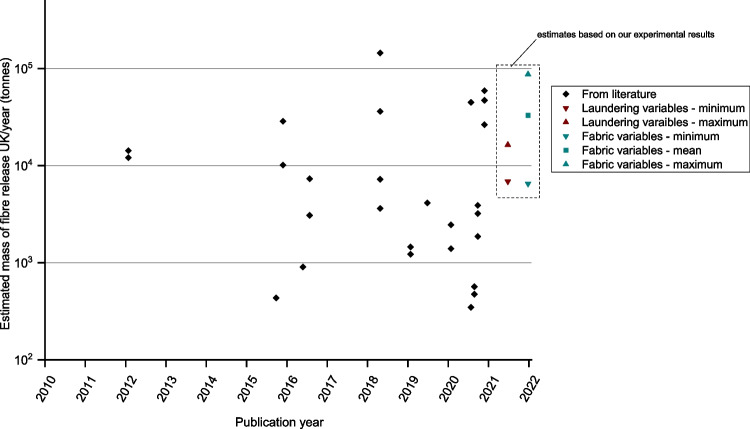
Comparison of the estimated mass of microfibre release UK/year (tonnes) including estimate based on our experimental results (logarithmic scale)

Some of these estimates are shown in Fig. 7 to compare our findings with other studies. In this figure, the minimum and maximum estimates for laundry variables from Table 4 are included, as are the minimum, maximum and mean estimates from the fabric variable testing.

## Conclusions

There have been a large number of studies investigating microfibres released from domestic laundry as an important source of ocean microfibre pollution. However, due to the multitude of different testing methods, laundering variables, and types of fabrics used for testing, there is a very wide range of estimates to quantify the scale of this pollution.

Considering the three major factors proposed here to influence the quantification of microfibre release—testing methodology, laundering variables, and fabric characteristics—the greatest spread of results is seen from differences in testing methodology as this includes different test methods, different laundering conditions, and different test fabrics. By normalising some of the published data on microfibre release and using assumptions for the size and number of wash loads, comparisons can be made for an indicative UK scenario for per annum microfibre release; estimations range from 17,617 billion microfibres or 347 tonnes to 2,602,080 trillion microfibres or 144,560 tonnes.

The influence of laundering and fabric variables was investigated using a standardised test methodology. Reduced liquor ratio, reduced agitation, and increased temperature, all resulted in a significant reduction in microfibre release. Microfibre release was also reduced with repeated washing, but a plateau was reached after the third wash. Fabric variables had a greater influence on microfibre release than laundering variables, however, no single fabric variable appeared to have a dominant influence, demonstrating the complexity of competing factors for different fabric types. Estimates for the UK-based scenarios described range from 6490 tonnes to 87,165 tonnes of microfibre release for the conditions tested.

## Data Availability

Not applicable.
